# Comparative Evaluation of Sucrosomial Iron and Iron Oxide Nanoparticles as Oral Supplements in Iron Deficiency Anemia in Piglets

**DOI:** 10.3390/ijms22189930

**Published:** 2021-09-14

**Authors:** Rafał Mazgaj, Paweł Lipiński, Mateusz Szudzik, Aneta Jończy, Zuzanna Kopeć, Adrian M. Stankiewicz, Marian Kamyczek, Dorine Swinkels, Beata Żelazowska, Rafał R. Starzyński

**Affiliations:** 1 Department of Molecular Biology, Institute of Genetics and Animal Biotechnology PAS, 28-130 Jastrzębiec, Poland; r.mazgaj@igbzpan.pl (R.M.); m.szudzik@igbzpan.pl (M.S.); a.jonczy@igbzpan.pl (A.J.); z.kopec@igbzpan.pl (Z.K.); adrianstankiewicz85@gmail.com (A.M.S.); b.zelazowska@igbzpan.pl (B.Ż.); 2 Pig Hybridization Centre, National Research Institute of Animal Production, 43-246 Pawłowice, Poland; marikamy@wp.pl; 3 Department of Laboratory Medicine (TLM 830), Radboud University Nijmegen Medical Center, 6525 GA Nijmegen, The Netherlands; dorine.swinkels@radboudumc.nl; 4 Hepcidin Analysis, Department of Laboratory Medicine, Radboud University Medical Center, 6525 GA Nijmegen, The Netherlands

**Keywords:** sucrosomial iron, absorption, erythropoiesis, iron deficiency anemia, iron supplement, pig, iron nanoparticles

## Abstract

Iron deficiency is the most common mammalian nutritional disorder. However, among mammalian species iron deficiency anemia (IDA), occurs regularly only in pigs. To cure IDA, piglets are routinely injected with high amounts of iron dextran (FeDex), which can lead to perturbations in iron homeostasis. Here, we evaluate the therapeutic efficacy of non-invasive supplementation with Sucrosomial iron (SI), a highly bioavailable iron supplement preventing IDA in humans and mice and various iron oxide nanoparticles (IONPs). Analysis of red blood cell indices and plasma iron parameters shows that not all iron preparations used in the study efficiently counteracted IDA comparable to FeDex-based supplementation. We found no signs of iron toxicity of any tested iron compounds, as evaluated based on the measurement of several toxicological markers that could indicate the occurrence of oxidative stress or inflammation. Neither SI nor IONPs increased hepcidin expression with alterations in ferroportin (FPN) protein level. Finally, the analysis of the piglet gut microbiota indicates the individual pattern of bacterial diversity across taxonomic levels, independent of the type of supplementation. In light of our results, SI but not IONPs used in the experiment emerges as a promising nutritional iron supplement, with a high potential to correct IDA in piglets.

## 1. Introduction

Iron deficiency is the most common mammalian nutritional disorder in the neonatal period. However, among mammalian species neonatal iron deficiency anemia (IDA), the most severe consequence of iron scarcity, occurs regularly only in pigs (*Sus scrofa domestica*) [1,2,3]. In the pig industry, the supplementation of piglets (on days 3–6 postpartum) with large amounts of iron dextran (FeDex) is a routine veterinary procedure preventing the development of IDA [4], but is connected with several side effects including, in some cases, the sudden cardiovascular collapse and respiratory failure [5]. On the other hand, oral administration of iron may be associated with potentially dangerous intestinal disorders such as diarrhea, abdominal pain, and constipation. It is well known that iron overdose may cause severe corrosive lesions to the upper gastrointestinal tract, such as necrosis of the mucous, ulcer, and ischemia [6]. Oral iron supplementation may lead to an imbalance in intestinal microbiota, that can influence the absorption of not only iron but also other micronutrients [7].

Oral supplements are commonly used for maintaining iron stores in humans [6]. Nevertheless, piglets’ supplementation with iron salts and chelates seems to be relatively inefficient [4,8,9], except when a significant dose of iron has been used [10]. Bioavailability of iron contained in supplements is a main determinant of their efficacy in combating IDA [11]. In newborn piglets, low bioavailability of inorganic (ionic) iron is due to a lack of iron transporters in the duodenum in early neonatal life [12]. For this reason, recently, we have used hemoglobin as a dietary source of heme, i.e., highly bioavailable organic iron, and found that it efficiently counteracts the development of IDA in piglets. In this study, we have investigated whether iron nanoparticles (IONPs) and Sucrosomial^®^ Iron (SI), which are believed to bypass classical pathways of iron absorption, would be an effective compound in the treatment of IDA in newborn piglets. IONPs have become a powerful tool for several biomedical [13] and environmental applications [14]. It is also well documented that IONPs can act as potential drugs and/or gene carriers, as well as contrast agents or hyper thermal mediators in anticancer therapy [15]. However, little is known about the usefulness of IONPs in the treatment of IDA. On the other hand, IONPs have been shown to be highly bioavailable [16] and nontoxic [17]. Sucrosomial iron (SI) represents an innovative oral iron-containing carrier, in which ferric pyrophosphate is protected by a phospholipid bilayer membrane mainly from sunflower lecithin and sucrester matrix [18]. Sucrester is a surfactant derived from the esterification of fatty acids with sucrose, which has recently been shown to behave as an absorption enhancer because of its ability to reduce intestinal barrier resistance. This formula ensures its high bioavailability and tolerance not only in rodents and humans [18,19], but also in pigs [20,21]. The efficacy of SI in correcting iron deficiency is attested by the restoration of physiological hemoglobin levels and has been proven in human and animal studies [19,22].

Here, we provide evidence showing that the oral supplementation of piglets with SI is at least as efficient in curing/preventing neonatal iron deficiency as a parenteral supplementation with FeDex routinely practiced in the swine industry. Although the application of SI and IONPs to piglets does not fully build up the hepatic iron stores of these animals compared to FeDex administration, it does, however, result in lower toxicity. To our knowledge, this study provides the first demonstration of a preventive/therapeutic use of IONPs and for SI in mammalian neonates, suggesting that, owing to its specific chemical formula, the iron contained in these compounds can be efficiently transported across the intestinal barrier and thus become bioavailable despite the immaturity of molecular mechanisms of iron absorption. Based on the results obtained in the present study, and considering the similarities between pigs and humans in terms of their gastrointestinal physiology [23], we suggest that the pig model is suitable for testing the usefulness of these and other iron supplements for preventing IDA in humans in early postnatal life. 

## 2. Results

### 2.1. Piglets Supplemented with Sucrosomial Iron^®^ (SI) but Not Iron Oxide Nanoparticles (IONPs) Show No Evidence of IDA

Oral supplementation of piglets with SI and FeSO_4_ (daily dose of 6 mg Fe) from day 5 up to weaning only partially resulted in maintaining values of red blood cell indices of 28 day-old animals at physiological levels (Table 1). 

Importantly, the hemoglobin concentration in these piglets (7.88 ± 0.63 g/dL) was close to the threshold values for anemia in pigs, i.e., 8 g/dL [24], 8.84 g/dL [25]. All other hematological parameters in the SI group were close to the normal ranges, but in the FeSO_4_ group, parameters such as MCV or MHC could indicate symptoms of anemia. Importantly, all nano-iron supplemented animals showed hematological values below the physiological range established for piglets at this age. In general, in piglets receiving SI only, values of other hematological parameters were similar (i.e., showed no statistically significant differences) to those evaluated in animals given parenteral injection with FeDex, i.e., the supplementation was considered as highly efficient in preventing the occurrence of IDA commonly used in pig rearing [4,25]. For control animals receiving no additional iron supplementation, all blood hematological parameters were below the reference data for this age group, showing signs of acute or moderate anemia [25]. Importantly for pig breeding and future fattening, although the initial birth weight was similar, the differences became apparent after 23 days of the experiment. A statistically significant difference was recorded in the case of animals who were parenterally administered iron in comparison to anemic animals. This translates into a daily weight gain for piglets orally supplemented with iron, which indicates the slow growth of piglets in relation to piglets injected with FeDex (Supplementary Table S1).

### 2.2. Piglets Supplemented with SI but Not IONPs Show Increased Blood Plasma Iron Level Compared to Anemic Animals although Iron Supplementation Does Not Increase Hepatic Iron Content 

**Figure 1:**
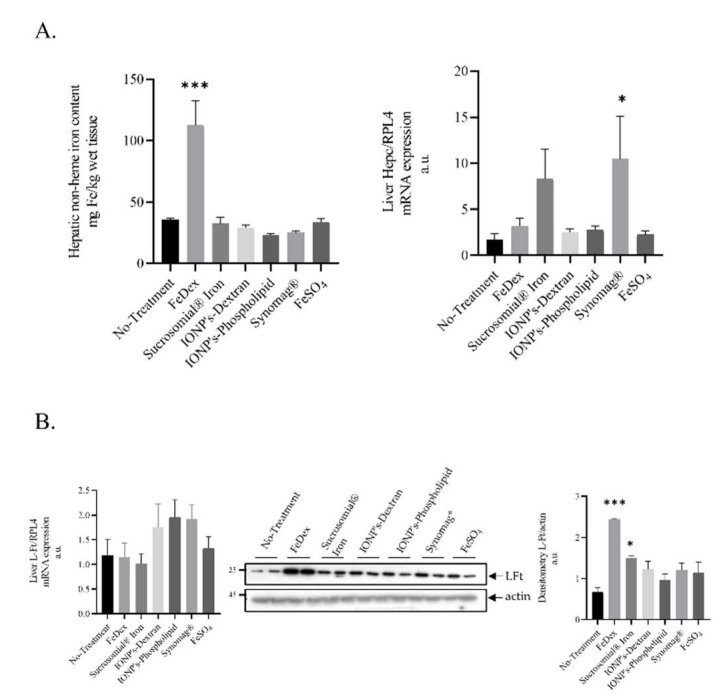
Animals supplemented with FeDex but not SI or IONPs show increased hepatic non-heme iron level and iron deposition in hepatic ferritin: (**A**): Non-heme iron in liver of 28 day-old piglets and hepatic Hepcidin mRNA level. Non-heme iron content and Hepcidin mRNA level was measured in liver as described in the Materials and Methods section. Analysis conducted on 42 piglets allocated into seven experimental groups (*n* = 6 per group). (**B**): The figure shows L-FT mRNA expression, representative Western blot, and the densitometric analysis of L-Ft protein levels. The samples were normalized for protein loading by actin. The data are expressed as arbitrary units obtained analyzing the bands by using the software Quantity One 4.6, Bio-Rad. Data are the mean ± SEM of *n* = 3 independent experiments carried out on 6 subjects for each experimental group. Data were analyzed with unpaired Student’s *t* test, * and *** asterisks denote statistically significant differences at *p* < 0.05 and *p* < 0.001 in comparison to control, non-supplemented animals.

To compare the iron status of piglets from all experimental groups, we performed a comprehensive analysis of iron content in their livers and spleens, as well as for the iron concentration in blood plasma. We found that plasma iron level in piglets supplemented with SI is almost two-fold higher compared to anemic animals (Table 2), which attests to the high nutritional bioavailability of SI-derived iron and denotes an adequate iron provision for erythropoietic demand, which is supported by the high reticulocyte count in animals from this group (Table 1). After the application of other oral iron supplements, the plasma iron level showed symptoms of hypoferremia. Similarly, the low reticulocytes count indicates a reduced supply of iron for erythropoietic needs (Table 1). In contrast to FeDex-derived iron, iron absorbed from oral supplements is not preferentially stored in the liver, as evaluated by the quantitative analysis of hepatic non-heme iron content (Table 2, Figure 1A). Preferential iron deposition from FeDex in the liver is also reflected in the protein level of light ferritin subunit (LFt) (Figure 1B). In the spleen, we detected no significant differences in non-heme iron levels between SI or IONPs and FeDex-supplemented animals (Table 2). Unexpectedly, we found high splenic iron content in anemic piglets.

### 2.3. Toxicological Markers Are Not Altered Neither in SI- Nor IONPs-Supplemented Piglets Compared to Control Animals

**Figure 2:**
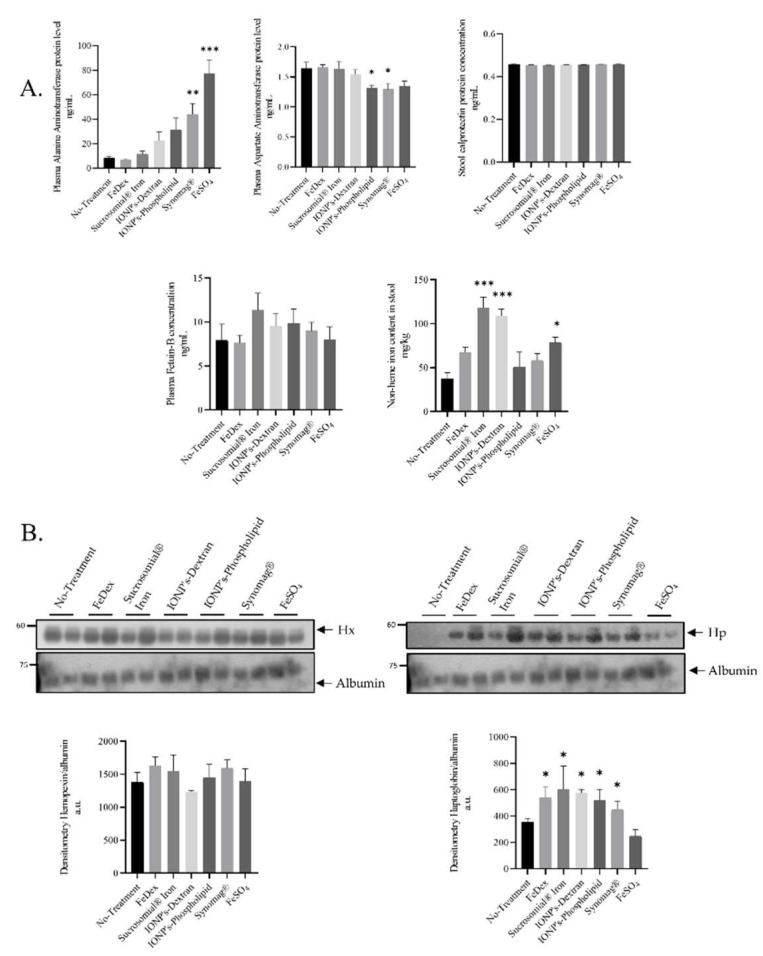
Toxicological markers do not alter in SI or IONPs-supplemented piglets compared to control animals: (**A**): Levels of toxicological markers: Aspartate aminotransferase (AST), alanine aminotransferase (ALT), and FetuinB were evaluated by ELISA using plasma from 28 day-old piglets. Calprotectin protein level was analyzed by ELISA from feces samples according to manufacturer protocol. The non-heme iron content in feces was measured as described in the Materials and Methods section. Analysis conducted on 42 piglets allocated into seven groups (*n* = 6 per group) (**B**): Hemopexin and Haptoglopbin plasma protein levels was measured using Western blot and the densitometry analysis was shown. The samples were normalized for protein loading by actin. The data are expressed as arbitrary units obtained by analyzing the bands using the software Quantity One 4.6, Bio-Rad. Data are the mean ± SEM of *n* = 3 independent experiments carried out on 6 subjects for each experimental group. Data were analyzed with unpaired Student’s *t* test, * and ** or *** asterisks denote statistically significant differences at *p* < 0.05 and *p* < 0.01 or *p* < 0.001 in comparison to control, non-supplemented animals.

The oral application of supplemental iron to suckling piglets raises the question as to the toxic effects of early life exposure to high iron in fodder. Considering that excess iron induces the production of reactive oxygen species and inflammation, we determined the level of calprotectin in the stool of 28 day-old piglets (Figure 2A), a reliable biomarker of intestinal inflammation [26]. The amount of calprotectin reflects the number of neutrophils participating in the inflammation. This has been widely confirmed in inflammatory bowel diseases through the significant correlation between fecal calprotectin levels and other hallmarks of acute inflammation [27]. We found that, in feces of either SI or IONPs-supplemented piglets, the calprotectin level was unchanged (Figure 2A). 

Fetuin-B is an acidic glycoprotein belonging to the Fetuin family and is a well-known negative acute phase protein. Fetuin-B, being a negative acute phase protein, takes part in the process of deactivating macrophages to restrain the innate immune response [28]. In plasma of piglets receiving SI and IONPs per os, we did not observe any changes in fetuin-B concentration, although upregulation in the SI-supplemented group was visible, albeit not statistically significant. Such an increase in fetuin-B levels may be due to the high levels of iron detected in the stool (Figure 2A), which then may contribute to the irritation of the gastrointestinal mucosa. The lack of oral supplementation or parenteral supplementation does not increase iron content in the stool, which translates into a low level of both calprotectin and fetuin B. We did not observe any changes in the plasma concentrations of transaminases Aspartate aminotransferase (AST) and alanine aminotransferase (ALT) in any of the examined group of animals (Figure 2A), except in Synomag^®^ and FeSO_4_-treated animals, where elevated ALT levels may be indicative of liver damage [29]. Acute-phase proteins, haptoglobin, C-reactive protein, and serum amyloid A (SAA) can be used as useful biomarkers for analyzing the risk of exposure to nanomaterials and associated toxicity [30]. Some data strongly suggest that hemopexin may serve as a biomarker for the analysis of biological responses related to an exposure to silica nanoparticles smaller than 100 nm [31]. Therefore, we decided to test hemopexin and haptoglobin serum levels in experimental piglets. We did not find any significant differences in the levels of hemopexin in iron-supplemented piglets compared to the controls, as shown by the Western blot analysis. In contrast, the densitometry of plasma haptoglobin bands revealed significant differences compared to controls in all iron-treated animals excluding FeSO_4_ supplementation (Figure 2B).

### 2.4. Barely Detectable Concentration of Hepcidin-25 in the Blood Plasma of Piglets Orally Supplemented with SI and IONPs

Hepcidin is an iron-regulatory hormone synthesized mainly by hepatocytes and secreted into the circulation as a 25 Amino-acids-long peptide that acts as a negative regulator of iron absorption from the duodenum and iron release from macrophages, recycling old red blood cells and thus adjusting iron supplies to the body’s iron requirements [32]. We have previously demonstrated that the measurement of hepcidin is helpful for guiding safe iron supplementation in piglets [20,33,34,35]. Here, we found that hepatic hepcidin mRNA is upregulated only in piglets supplemented with SI and Synomag^®^. This increase was not statistically significant in comparison to anemic animals (Figure 1A). Interestingly, concentrations of hepcidin-25 in the blood plasma of piglets supplemented orally with iron preparations or parenterally with FeDex are below the lower limit of detection, i.e., 0.5 nM (Data Not Shown).

### 2.5. FeSO_4_ but Not Other Oral Iron Supplements Increases Duodenal Hepcidin mRNA Abundance

**Figure 3:**
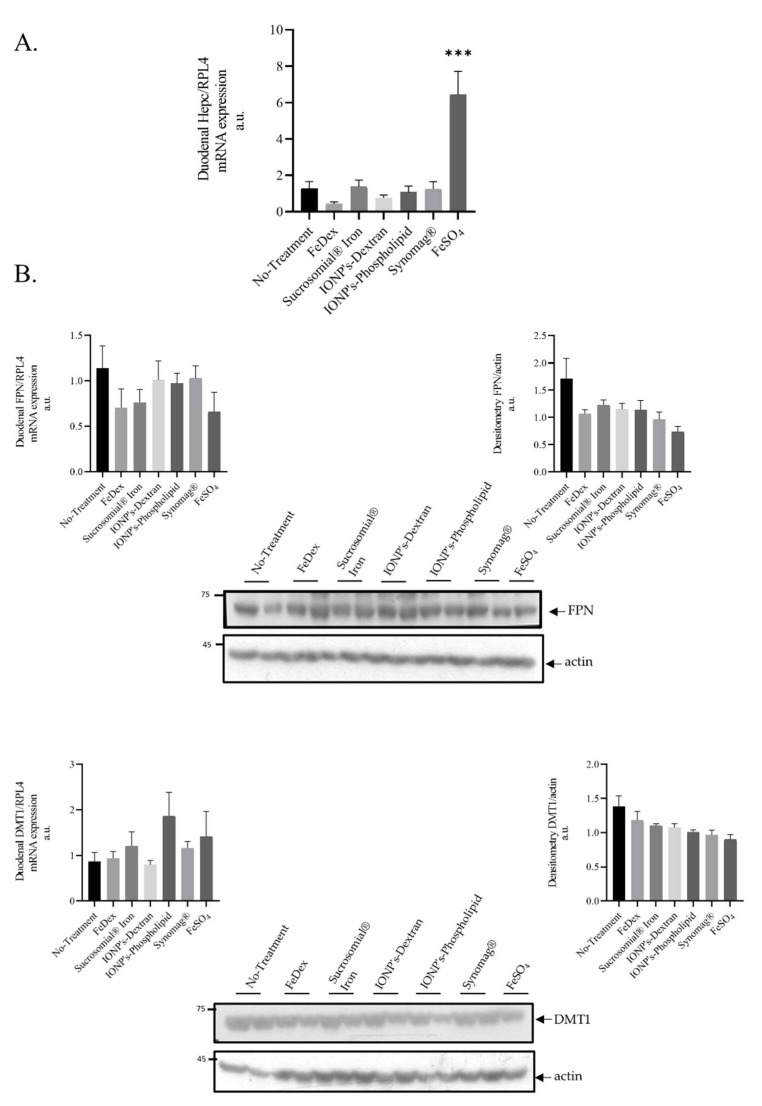
Oral FeSO_4_ administration but not Sucrosomial iron or IONPs increases duodenal hepcidin level without affecting duodenal iron transporters: (**A**): Duodenal Hepcidin mRNA expression in 28 day-old piglets (*n* = 6 per experimental group); (**B**): Analysis of duodenal iron transporters. Duodenal mRNA lof FPN and DMT1 levels and protein analysis by Western blot, as well as its densitometry, is shown. The samples were normalized for protein loading by actin. The data are expressed as arbitrary units obtained by analyzing the bands using the software Quantity One 4.6, Bio-Rad. Data are the mean ± SEM of *n* = 3 independent experiments carried out on 6 subjects for each experimental group. Data were analyzed with unpaired Student’s *t* test, *** asterisks denote statistically significant differences at *p* < 0.001 in comparison to control, non-supplemented animals.

It is well established that hepcidin is expressed locally in the intestine and, more precisely, in the dendritic cells present in the gut [36]. Therefore, we investigated the expression of local hepcidin levels in duodenal scrapings from orally iron-supplemented animals. The expression pattern of hepcidin mRNA in the duodenum of piglets treated orally and parenterally with iron supplements is completely different than that in the liver. We noted a strong increase in duodenal hepcidin mRNA level in FeSO_4_-supplemented piglets (Figure 3A). However, this increase did not affect the level of ferroportin (FPN), a molecular target of hepcidin and the sole iron exporter from duodenal enterocytes into the blood (Figure 3B). Similarly, no changes in the expression of divalent metal transporter 1 (DMT1), an iron importer from the intestinal lumen to the enterocyte, was noted (Figure 3B).

### 2.6. The Role of Oral Iron Supplementation in Modulation of Piglets Microbiome

Microbial colonization of the piglet gut begins immediately following birth. Here, we asked whether oral iron supplements and the mother would influence the diversity of gut microbiome of piglets.

**Figure 4:**
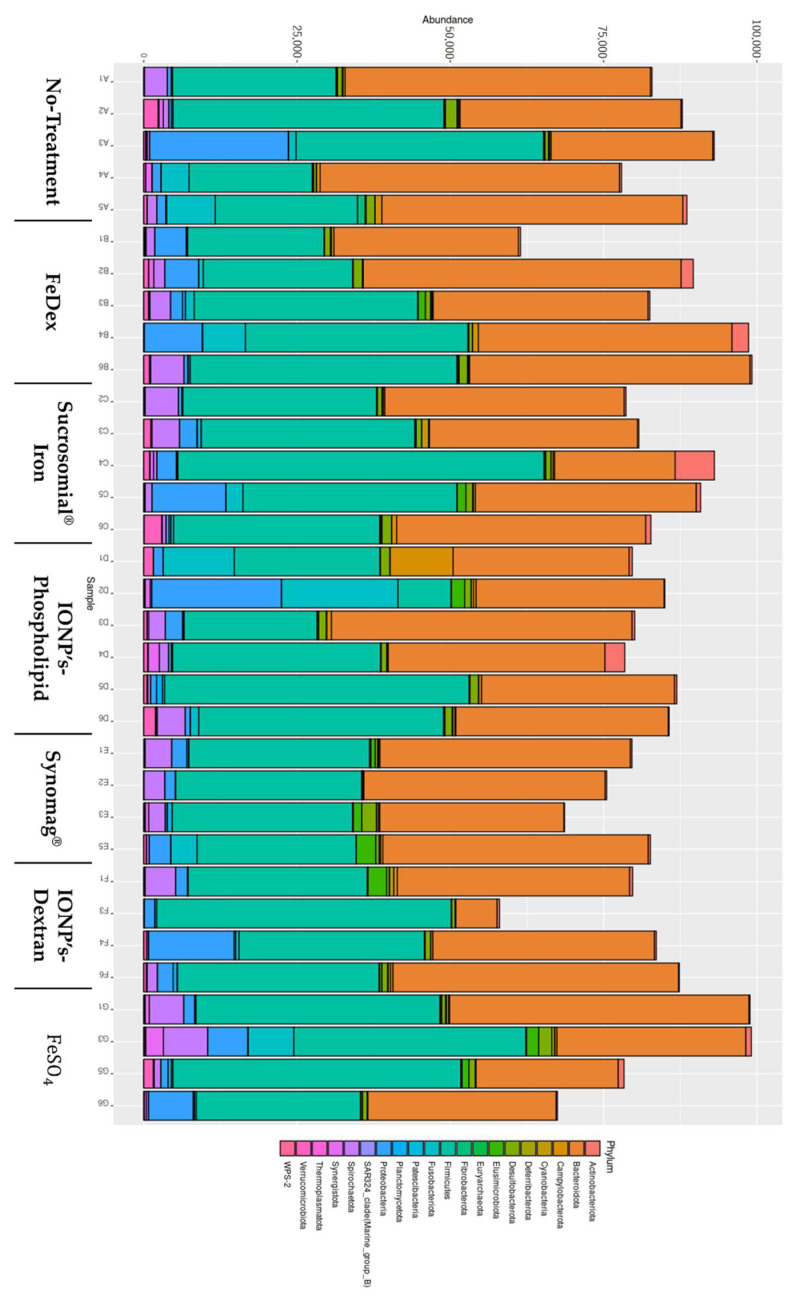
The role of oral iron supplementation in modulation of piglets’ microbiome. Microbiome abundance representation by Phylum.

First, we checked the relative abundance of taxons in our samples. An abundance of the phylum level is shown in Figure 4, while full data is available in supplementary files. Of all the sequenced Amplicon sequence variants (ASVs), more than three-quarters belonged to two classes: Bacteroidia and Clostridia, and more than half belonged to six families: Prevotellaceae, Oscillospiraceae, Rikenellaceae, Muribaculaceae, Bacteroidaceae, Lachnospiraceae. Next, we asked whether iron supplementation and its type influences the diversity of the gut microbiome of piglets and can be affected by the mother. We found that intramuscular injection of FeDex decreases microbe diversity, as shown by both Shannon (*p* value 0.018) and Simpson (*p* value < 0.001) within- sample diversity indices (see supplementary Table diversity_statistics.xlsx-alpha_values (a)). For the No-treatment vs. FeDex comparison, DeSeq2 analysis using the Wald method showed 36 differentially abundant ASVs, with the three most significant belonging to Christensenellaceae R-7, Prevotella, and Butyricimonas genera (muscleDextran_diff_abund.xlsx). No effect of supplementation was found for DEICODE between- sample diversity metric (F value 0.653, *p* value 0.711, see supplementary Table diversity_statistics.xlsx-beta_values (a,b)). On the other hand, the mother affected microbiome between-sample diversity metric (F value 2.772, *p* value 0.027, see supplementary Table diversity_statistics.xlsx-beta_values (a,d)). To study this effect further, we updated our dataset by removing a single sample originating from piglets born to a mother that had no representation in other supplementation groups. Next, we performed batch correction to remove the supplementation effect. The following results are based on the updated dataset. Because, currently, the betta test allows only for pairwise comparisons, we contrasted within-sample diversity indices of offspring of the first mother (id 2983_15) to those of the offspring of the other mothers and found significant differences according to both metrics. This suggests that the mother affects within-sample diversity of piglets’ fecal microbiome. Similarly, we found a significant maternal effect in the PERMANOVA test for differences in between-sample diversity (F value 3.81, *p* value 0.01, see supplementary Table diversity_statistics.xlsx-beta_values (b,e)). Using pairwise PERMANOVA, we found several significantly differing mother pairs, though none survived adjustment for multiple comparisons. The mother accounted for a large portion (42%) of total between-sample diversity variance. To visualize the similarity between the samples, we plotted DEICODE distances between them on a PCoA graph (Supplementary Figure S1). The visualization shows a clear separation of samples into two clusters. The bulk of the smaller cluster (S) is composed of nearly all samples from mother 5142 and the larger one (L) are the samples from mothers 5201, 2983, and 6838. Different samples from mothers 4283 and 7081 fall into either of these clusters. Finally, we performed differential abundance analysis to identify the ASVs that vary the most between mothers. We were interested only in the mothers’ effect in general and not in any particular comparison between them. Hence, we performed the analysis using an LRT method, which looks for an effect of given variable, irrespective of specific contrasts. Results are available in supplementary data (mother_diff_abund.xlsx). Only two ASVs were significantly and differentially abundant after adjustment for multiple comparisons. These ASVs correspond to an unknown species of Terrisporobacter genus and Clostridia class. 

As mentioned previously, we found that samples group into two clearly separate clusters (Supplementary Figure S1). We decided to study this further using the original dataset with all samples intact. The cluster effect was highly significant, according to PERMANOVA analysis (*p* value < 0.001) (cluster_analysis.xlsx), and explained 72% percent of sample variation. A total of 206 differentially abundant ASVs were identified (cluster_analysis.xlsx). Thus, we speculate that there exists an unknown factor switching the microbiome pattern of piglets between two characteristic states.

## 3. Discussion

Hepatic iron stores are relatively low in newborn piglets [12,37]. We have previously reported a drastic decrease in iron content in the liver (to a barely detectable level) of the Polish Landrace piglets between day 1 and 3 postpartum [33]. The fortification of iron status using exogenous iron to prevent/treat iron deficiency in weanling piglets is of great importance. Intramuscular bolus injection of high amounts of iron in the form of FeDex to piglets is a traditional way to counteract against iron deficiency in pig breeding [1,3,4]. It has also been well established that oral supplementation with iron is usually much less effective than the parenteral one [8,9,24], unless iron is given per os in excessive quantities (grams of Fe/kg of fodder) [10]. The absorption of iron from oral supplements is a complex process, in which several iron transporters and other regulatory proteins are involved at the apical and basolateral membrane of duodenal enterocytes [38]. Moreover, poor responsiveness of newborn to orally administered iron salts and chelates is caused by the immaturity of the digestive tract and is associated with a very low expression of crucial iron transporters in the duodenum [12]. Nevertheless, the oral route of iron administration to piglets still remains potentially an attractive procedure as it is noninvasive. Nanotechnology is a field of research offering innovative and promising products that, among others, have been recently used to generate nutrients with increased bioavailability. Moreover, since IONPs are the only magnetic nanomaterials approved for clinical use by the US Food and Drug Administration [39], it is essential to determine their biocompatibility for in vivo biomedical applications to ensure their safety in clinical trials [40]. Many studies have shown that, when materials are prepared in nanometer size, their bioavailability significantly increases [41]. Similar to zinc phosphate-based nanoparticles, a zinc supplement [42], it is believed that, with a size reduction of iron containing compounds to nanometer size, iron bioavailability will also increase. In addition to nanoparticles, liposomes (small vesicles enclosed by a lipid bilayer membrane) are widely used as carriers for drugs that can reduce systemic toxicity and increase drug delivery to the target sites in the body [43]. Importantly for this study, iron compounds encapsulated in liposomes have been successfully used to relieve the iron deficiency in various animal models [44]. Here, we used pure nanoparticulate iron and iron encapsulated in organic materials, such as dextran and lipids (lecithin), to increase the stability of nanoparticles in a biological environment, especially to minimize contact of IONPs with the hydrochloric acid of the stomach and finally the formation of soluble iron salts, which are actually the ionic iron. We also used Sucrosomial^®^ iron, a liposomal iron formula based on Sucrosomial^®^ technology [18], to compare the efficacy of liposomes encapsulated iron salt with encapsulated iron oxide nanoparticles in combating neonatal IDA. The indication for this comparison in neonatal IDA in the pig model is based on our previous pilot study showing that the supplementation of suckling piglets with Sucrosomial^®^ iron during a period of 23 consecutive days (from day 5 to day 28 after birth) results in a similar therapeutic effect and decreases toxicity compared to the injection iron dextran on day 3 after birth [20]. The evaluation of the preventive/curative effect of piglets’ supplementation with SI was based on a comparison between the parenteral treatment of piglets with FeDex, a procedure with proven efficacy and which is widely used in pig rearing [4], and with the supplementation with ferrous sulphate, considered to be the “golden” standard for oral iron supplementation. 

Recent findings published by Pereira et al. clearly show that nanoparticulate iron(III) oxo-hydroxide delivers safe iron that is well absorbed and utilized in humans [45]. Due to their physicochemical properties, IONPs are far more effective than heme and non-heme iron microparticulate in the correction of iron deficiency anemia in piglets, as has been demonstrated by Churio et al. [46]. Wegmüller et al. [47] assessed the effect of size reduction and the encapsulation of iron pyrophosphate on hemoglobin retention in anemic rats and reported that the bioavailability of iron pyrophosphate with a mean size of 2.5 μm was 43%, and that with the size of 0.5 μm, it was 95%, respectively, compared to ferrous sulfate. In our study, only animals supplemented with FeDex, SI, and FeSO_4_ showed values of RBC indices within the physiological range established for this age group of pigs (30 days old) supplemented on days 3–5 with a single injection of iron dextran [24,25]. Interestingly, all RBC parameters from IONPs-supplemented piglets did not differ significantly from those in anemic animals. Importantly, physiological RBC status of piglets receiving SI as well as IONPs was accompanied by their normal growth performance, as their bodyweight gain during the first 4 weeks of life met the growth standards for the Polish Landrace breed and was higher than in anemic piglets. Our results prove that the amount and bioavailability of iron provided with SI and, to a certain degree, with lipid-encapsulated IONPs, are adequate for meeting the erythropoietic needs of suckling piglets. As shown previously [20], in the case of SI-treated animals, oral supplementation resulted in increased blood plasma iron level compared to control (anemic) animals. Contrary to other authors who have shown that single dose of nanoparticles had a higher bioavailability and better improved RBC indices in rat hemolytic anemia compared to ferrous sulfate [48], in our experiment, we did not see any curative effect of IONPs in anemic piglets. The opposite of our results was obtained using nano-iron by hot melt extrusion (HME, 100 ppm Fe) as an alternative to ferrous sulphate. In many studies, the feed concentration of iron contained in nano particles ranges from 100 to 350 mg/kg feed [49,50]. It has been shown that the dietary nano-iron supplementation does not significantly improve the growth performance of weanling pigs [50]. Similarly, in our experiment, the growth of IONPs-supplemented piglets in the first days of life was relatively high but not significantly different from anemic animals, in which significant growth stunting was observed in comparison to FeDex-supplemented piglets. Such a problem does not exist with parenteral administration.

The appearance of the first symptoms of IDA is usually preceded by the reduction of hepatic iron stores without a decline in the RBC count. Therefore, we evaluated the liver iron content of piglets supplemented with SI or IONPs and found that animals from both groups showed no excessive iron accumulation to FeDex-treated piglets. Lower hepatic iron content in piglets supplemented with SI or IONPs compared to those given FeDex may be due to the fact that, after an intramuscular injection of FeDex to animals, it enters the reticuloendothelial cells (RES) in the liver and thus contributes to the excessive iron accumulation in this tissue [51]. By saving RES from iron overload, supplementation with SI or IONPs does not contribute to the induction of hepcidin expression [52], a peptide that, in newborn piglets, inhibits the intestinal absorption of inorganic iron through the own-regulation of ferroportin expression [12,34,35]. Animals supplemented with both SI and IONPs showed no evidence of increased blood plasma hepcidin concentration, which remained below the lower limit of detection, i.e., 0.5 nM (data not shown). The expression of hepcidin is induced transcriptionally, mainly in response to iron. There is experimental evidence suggestive of the fact that both hepatic iron loading [53] and high saturation of plasma transferrin with iron [54] and stimulate hepcidin synthesis. We found a slight hepatic upregulation of the hepcidin mRNA level in the Synomag^®^ group of animals compared to the anemic group; however, in piglets supplemented with SI or IONPs, neither transferrin saturation nor the hepatic iron level reached a threshold indispensable for increasing the blood plasma hepcidin level. We also found a very low blood plasma hepcidin level (below 0.5 nM) in FeDex-treated animals. By contrast, hepcidin-25 concentration was strongly elevated in the plasma of 28-day-old piglets subjected to the procedure of two-fold FeDex injection on days 3 and 21 after birth, as used in our previous studies [33,34,35]. Moreover, we have found a high splenic iron level in the anemic group of animals. This phenomenon may be explained by the fact that iron recirculation by the spleen is reduced at the expense of iron delivery for locally occurring erythropoiesis. Accordingly, during neonatal period, when the bone marrow is not yet fully responsible for erythropoiesis, the spleen is one of extra marrow soft tissues that produces red blood cells [55]. Upon SI treatment, we also found an elevated iron level in blood plasma, which is a source of iron for erythropoiesis, as it is known that diferric transferrin in the blood plasma is the major molecule providing iron for erythroid heme synthesis [56].

According to the double-edge sword nature of iron, this biometal, apart from being an essential micronutrient, is potentially toxic due to its redox properties leading to the generation of highly toxic reactive oxygen species. An important aspect in the biomedical application of IONPs is that their structure ensures low toxicity and small innate bioreactivity. Number of short- and long-term studies using numerous biocompatibility and stability tests, e.g., the hemolysis test, micronucleus assay, detection of median lethal dose [57], cell viability, glutathione and ROS production [58], degradation and biotransformation over time assessment in vivo [59] have all showed IONPs’ low and short-term toxicity and absence of abnormalities or mutations in the long term. It has been well established that the administration of iron to piglets in the form of FeDex carries a risk of over supplementation. In this procedure, piglets are usually injected with an amount of iron which exceeds 4–5 times the total content of this microelement in their body. We have previously demonstrated that the supplementation of piglets with FeDex induces oxidative damage to DNA in the liver [12]. Here, we report that piglets supplemented with daily dose of 6 mg Fe in the form of SI or IONPs showed the plasma concentration of fetuin B (the biomarker of liver fibrosis [60]) as well as that of aminotransferases AST (liver damage markers and predictor of liver function) at a level similar to control animals. Although, in this study, we cannot calculate the exact amount of iron absorbed by piglets from SI or IONPs, our observation showing the relatively high iron content in the feces of piglets from SI and Synomag^®^ groups suggests that a part of iron applied orally was not absorbed by the piglets. In this context, it is worth noting that the level of fecal calprotectin, a biomarker of the inflammation of the alimentary tract, shows no changes in any of the supplemented groups. This result may be related to the low total amount of daily-given supplemental iron, including non-heme iron (as measured in the stool of piglets from all examined groups), which may potentially be an irritant for the intestine. Importantly, second aminotransferase, namely ALT, was significantly elevated in serum from piglets receiving pure iron oxide nanoparticle and pure iron salt. In contrast to our data, the potential pro-inflammatory and allergenic effects of IONPs have been shown in inhalational administration [61]. Mice intratracheally administered with IONPs demonstrated elevated levels of many inflammatory cytokines (IL1, HSF1, TNFα) over a period of 28 days. This study provides evidence against inhalational but not oral use of IONPs. A single dose of nanoparticles displayed greater bioavailability compared to ferrous sulfate. Furthermore, the two doses of nanoparticles caused lower inflammation than one dose of ferrous sulfate [48]. Similarly, some minor toxic effects have been observed in the human intestinal Caco-2 cell line as well as in in that of rats. The nanomaterial was found to have minimal cytotoxic effects on the cells and no undesirable effects were reported on the intestinal mucosa and fecal microbiota of the rats [45]. According to the results produced by Elsayed et al. [49], magnetite and folate-coated magnetite nanoparticles can induce their toxic effects on different organs and abnormal fluctuations of some parameters of the blood picture occur within a 3-week timeframe in the rats. Studies using nanomaterials suggest that haptoglobin (Hp), C-reactive protein (CRP), and serum amyloid A (SAA) are highly sensitive biomarkers for assessing the risk of exposure to silica nanoparticles [30,31]. Our results indicate a higher incidence of oxidative stress and inflammation, as measured by serum haptoglobin levels, in iron-supplemented piglets compared to control animals, similar to the results obtained using intravenously injected mice with silica nanoparticles [30]. On the other hand, we did not detect any changes in the hemopexin levels of piglets receiving the oral iron supplementation in comparison to control animals. Hemopexin (Hx) is another acute phase protein that, along with Hp, is induced during infection and after inflammation to minimize tissue damage and facilitate tissue repair [62]. In contrast, quantitative image analysis showed higher levels of hemopexin in mouse plasma after an administration of 70 nm silica particles [30]. Both Hp and Hx are acute-phase proteins released from the liver upon the action of inflammatory cytokines. It is also conceivable that, instead of inflammatory cytokines, some part of iron nanoparticles act directly on the liver to induce the release of acute-phase proteins. However, a daily dose of 6 mg of iron did not induce any significant elevation of liver injury (micro and macroscopic analysis) or dysfunction markers, such as ALT or AST. Therefore, it is unclear why iron supplementation triggers the production of haptoglobin. 

Our hypothesis regarding the well-known phenomenon of high bioavailability of SI or IONPs-derived iron relies on the assumption that these compounds enter the circulation from the duodenal lumen through apical and basolateral membranes in the intact form without the involvement of any duodenal transporters. Indeed, we have shown that the expression of both duodenal iron transporters DMT1 and ferroportin were not influenced (changed) by the administration of supplemental iron in comparison to the control piglets. It is possible that iron contained in SI or IONPs can move through the duodenum barrier independently of molecular mechanisms of iron absorption, which are immature in newborn piglets [12,33]. After being transferred to the blood, a large number of intact IONPs or SI is trapped by RES macrophages in the liver and spleen. Ex-vivo permeation experiments, carried out using the excised rat intestine model, have shown that the presence of sucrester protects trivalent pyrophosphate iron in SI against enzymatic reduction and promotes its absorption across the intestinal epithelium through a DMT1 independent pathway [63]. Moreover, the epiintestinal human 3D tissue models have confirmed the presence of vesicle-like structures during the intestinal absorption of SI and its different absorption kinetics, compared to ferrous sulfate and ferrous bisglycinate [63]. Interestingly, in the case of Sucrosomial^®^ iron, it has been suggested that, similar to all sucrosomes, this iron-containing particle is absorbed through M cells (microfold cells) that are present throughout the gut-associated lymphoid tissue (GALT) of the Peyer’s patches and in the mucosa-associated lymphoid tissue (MALT) of other parts of the gastrointestinal tract [64]. The possible role of an M cell-mediated pathway in SI absorption was investigated using an in-vitro CACO2/RajiB co-culture system, where the presence of M cells (RajiB cells) enhanced SI absorption, followed by passing through M cells SI was taken up by CD68+ macrophages [65]. Possibly, digestion and decomposition of SI and IONPs in RES macrophages is followed by the release of iron and its recirculation into the bloodstream and is then transported to the sites of its bio utilization, mainly in the bone marrow for heme synthesis. 

One of the criteria which should be considered in the case of oral iron supplementation is its influence on gut microbiome. It is worth noting that, for example, per os iron fortification with iron salts adversely affects the gut microbiome, increasing pathogen abundance and inducing intestinal inflammation in Kenyan children [66]. Food additives such as iron oxides and hydroxides (E172) induce gastrotoxicity, hepatotoxicity, and alterations in gut microbiota, and most evidence points to oxidative stress as the main mechanism of toxicity [67]. However, several human studies have shown that nano Fe(III) was found to be a better alternative, having no negative impact on the gut microbiome [45]. Another study noted that iron nanoparticles increased the diversity and viability of the microbiota, marked by an increase in the number of bacteria belonging to the *Lactobacillus* genus. On the other hand, dietary iron depletion in weaning rats imprints low microbiome diversity [45]. These data encourage us to perform a molecular analysis of microbiota in piglets. 

The assessment of bacterial diversity was carried out using the NGS technology and bioinformatics analysis. The results of these studies indicate the individual-dependent differentiation of bacteria at particular taxonomic levels, regardless of the type of supplementation, which may be related to a large number of factors influencing the changes in the microbiome, such young age. Interestingly, regardless of the type of supplementation, a statistically significant maternal effect on the bacterial spectrum of the intestinal microbiota was demonstrated, which may indicate both a strong environmental influence as well as a genetic determination for the specific colonization of the intestine by bacteria. The microbial colonization of the piglets’ intestine begins immediately after birth or even in the reproductive tract [67], and the initial colonization by *E. coli* and *Streptococcus* spp. creates an anaerobic environment for the subsequent colonizers: *Bacteroides*, *Bifidobacterium*, *Clostridium*, or *Lactobacillus* [68,69,70]. From our results, we can conclude that the microbes that actually colonize the piglets’ intestinal tract depend on both the sow and the piglets’ environment, including their diet, and the readily bioavailable supplements administered to the microorganisms. Similar to others [71], we also found that the additional provision of iron did not change the ratio of Firmicutes/Bacteroides (F/B), which is indicative of the overall health of the gastrointestinal tract.

## 4. Materials and Methods

### 4.1. Piglets, Experimental Design and Biological Sample Collection

Experiment was conducted at the Pig Hybridization Centre in Pawłowice belonging to the National Research Institute of Animal Production (Balice, Poland). A total of 42 Polish Landrace piglets (males) from 7 different litters housed in standard conditions (70% humidity and a temperature of 22 °C in cages with straw bedding) were used. During the 28-day experiment, sows were allowed to nurse their piglets, and piglets had no access to the sows’ feed. The Prestarter Wigor 1 Plus (Wipasz, Olsztyn, Poland), feed (containing 238 mg Fe per 1 kg as estimated by flame spectrometry) was offered to piglets from day 5 to day 28 after birth. The composition and nutritive value of Prestarter Wigor 1 Plus is shown in Supplementary Table S2. Piglets from 7 different litters were randomly allotted to 7 experimental groups (groups made from one piglet from every sow) on the basis of balanced body weight and equalized red blood cell indices at day 3 after birth: (No-treatment) piglets without any iron supplementation, *n* = 6; (FeDex) piglets supplemented parenterally with 100 Fe mg/kg b.w. on day 3 postpartum, by intramuscular injections (in the neck) of iron dextran (FeDex), a complex of ferric ions with low molecular weight dextran (Ferran^®^100, Vet-Agro, Lublin, Poland, Fe^3+^ 100 mg/mL, supplementation routinely applied to piglets at the Pig Hybridization Centre in Pawłowice), *n* = 6; (Sucrosomial^®^ Iron) piglets supplemented per os from day 5 to day 28 after birth with a daily dose of 6 mg Fe in the form of Sucrosomial^®^ iron (PharmaNutra, Pisa, Italy) suspended in 2 mL of milk replacer; *n* = 6 (IONP’s-Phospholipid) piglets supplemented per os from day 5 to day 28 after birth with a daily dose of 6 mg Fe in the form of phospholipid (phosphatidynocholine) coated iron nanoparticle (iron oxide-lipid, Ø 100 nm, cat no 5 4119-5, Micromod Partikeltechnologie GmbH, Rostock, Germany); *n* = 6. (IONP’s-Dextran) piglets supplemented per os from day 5 to day 28 after birth with a daily dose of 6 mg Fe in the form of dextran coated iron nanoparticle (iron oxide-DX, Ø 50 nm, cat no 4104-5, Micromod Partikeltechnologie GmbH, Rostock, Germany); *n* = 6. (Synomag^®^) piglets supplemented per os from day 5 to day 28 after birth with a daily dose of 6 mg Fe in the form of pure nanoparticle (Synomag, Ø 30 nm, cat no 4104-5, Micromod Partikeltechnologie GmbH, Rostock, Germany); *n* = 6. (FeSO_4_) piglets supplemented per os from day 5 to day 28 after birth with a daily dose of 6 mg Fe in the form of ferrous sulfate (Gambit, Kutno, Poland); *n* = 6. 

The initial body weight (similar in all experimental groups) and the final body weight were monitored in all experimental groups (Supplementary Table S1). Blood was drawn on day 28 after birth by venipuncture of the jugular vein (Vena jugularis externa) into tubes coated with heparin as an anticoagulant. The blood samples were centrifuged (1500× *g*, 10 min, 4 °C) to separate plasma. Plasma samples were immediately aliquoted and stored at −80 °C. Twenty-eight day-old piglets were euthanized by the intracardiac injection of 0.5 mL\kg b.w. of Morbital (133.3 mg/mL of sodium pentobarbital + 26.7 mg/mL of pentobarbital; Biowet, Puławy, Poland). Tissue samples were collected, rinsed with PBS (Phosphate Buffer Saline) and then stored at −80 °C until they were used for biochemical analyses. Feces were collected directly from the anus postmortem. The proximal segment (5 cm) of the duodenum downstream of the stomach was dissected postmortem from piglets, carefully washed with PBS, and preserved for further analyses. The other part was further dissected to obtain a highly enriched epithelium fraction. A lancet was used to scrape the upper layer of the duodenum, making efforts to avoid the circular muscle. Duodenal scrapings were then stored at −80 °C until they were used for Western blotting and RT-qPCR analyses.

### 4.2. Measurement of Red Blood Cell Indices and Plasma Iron Parameters 

Hematological indices were determined using an automated IDEXX ProCyte Dx hematology analyzer (IDEXX Laboratories, Inc., Westbrook, ME, USA). The plasma iron concentration was determined by colorimetric measurement of an iron-chromazurol complex according to the manufacturer’s protocol (BioMaxima, Lublin, Poland). Total iron binding capacity (TIBC) was determined by colorimetric measurement of the absorbance of the iron-chromazurol complex at 630 nm according to manufacturer protocol (BioMaxima, Poland). Percent of transferrin saturation (TSAT) was then calculated according to the following formula: TSAT = (plasma iron/TIBC) × 100.

### 4.3. Measurement of Calprotectin in Feces and Plasma Aspartate Transaminase, Alanine Transaminase and Fetuin-B Levels

Level of calprotectin, the intestinal inflammation stress marker, was measured in feces using ELISA kit, according to the manufacturer’s protocol (Wuhan Fine Biotech Co., Ltd., Wuhan, China). Plasma aspartate transaminase (AST), alanine transaminase levels (ALT), and fetuin-B was measured by ELISA according to the manufacturer’s protocol (Wuhan Fine Biotech Co., Ltd., Wuhan, China). All intra- and inter-assay coefficients of variation were between ≤4.1 and 18.5.

### 4.4. Measurement of Iron Content in Tissues

The non-heme iron content in liver and spleen (100 mg) were determined by acid digestion of the samples at 100 °C for 10 min, followed by colorimetric measurement of the absorbance of the iron-ferrozine complex at 560 nm as described previously [72].

### 4.5. Blood Plasma Hepcidin-25 Quantification

Hepcidin-25 measurements were performed as described previously for porcine plasma samples [34,73] by a combination of weak cation exchange chromatography and time-of-flight mass spectrometry (WCX-TOF MS) using a stable hepcidin-25 + 40 isotope as internal standard for quantification. Peptide spectra were generated on a Microflex LT matrix-enhanced laser desorption/ionisation TOF MS platform (Bruker Daltonics, Billerica, United States). Hepcidin-25 concentrations were expressed as nmol/L (nM). The lower limit of quantification of this method was 0.5 nM. The concentration of pig hepcidin-25 was calculated by comparing its mass peak height with that of the internal standard [74].

### 4.6. Real-Time Quantitative RT-PCR

Total cellular RNA was extracted from tissues (20 mg) and cell pellets using Trizol reagent (Invitrogen) according to the manufacturer’s protocol. Two micrograms of total DNAse-treated RNA were reverse transcribed using a Transcriptor First Strand cDNA Synthesis Kit (Roche, Switzerland). Real-time quantitative PCR analysis was performed in a LightCycler 96 (Roche Diagnostics, Mannheim, Germany) using gene-specific primer pairs (Supplementary Table S3). The amplified products were detected using SYBR Green I (Roche Diagnostics) as described previously [73]. To confirm amplification specificity, the PCR products were subjected to melting curve analysis and agarose gel electrophoresis. LightCycler 96 Software was used for data analysis. Transcript levels were normalized relative to the control reference gene selected using NormFinder software [75]; (https://moma.dk/normfinder-software (accessed on 11 August 2021)).

### 4.7. Protein Extract Preparation and Western Blotting

For the analysis of proteins, crude membrane extracts were prepared from duodenal scrapings and cell pellets, as described previously [76,77], and cytosolic protein from liver tissue and cell cultures preparation have been shown by Drapier et al. [78] For Western blot analyses of FPN, TfR1 FtH, FtL, actin and DMT1. Laemmli sample buffer was added to 40 µg of protein samples and samples were processed according to the Laemmli SDS PAGE procedure on 9% or 14% gels, depending on molecular weight of the protein. To determine the plasma haptoglobin (Hp), hemopexin (Hpx), and albumin (Alb) levels, 5 μL for Hp and Hx and 1 μL for Alb samples of 40-fold diluted piglet plasma were boiled and resolved by electrophoresis on a 10% SDS/PAGE gels. Electroblotting of the resolved proteins on the PVDF membranes (Thermo Scientific, Waltham, United States), blocking and incubation with primary and secondary antibodies were performed. A list of used primary and secondary antibodies and their dilutions is shown in Supplementary Table S4.

### 4.8. Statistical Analysis

All the data except that from microbiome analysis were analyzed statistically with one-way analysis of variance (ANOVA), and Tukey–Kramer post hoc test using GraphPad Prism software (GraphPad, San Diego, CA, USA). *p* ≤ 0.05 was considered significant. Data are presented as mean values ± SEM. Experiments were designed in two replicates.

### 4.9. Metagenomics Analysis 

#### Data Analysis

Data analysis steps were performed in R [79,80], unless stated otherwise. All figures were drawn using ggplot2 from tidyverse package set [81]. MMUPHin R package was used for batch correction [82]. Shannon and Simpson alpha (within-sample) diversity indices were calculated using DivNet [83]. The indices reflect both richness and evenness of the studied sample. The DivNet method estimates true diversity of microbes in the given ecosystem based on the provided samples. It improves upon previous models of diversity estimation by accounting for both the positive and negative correlation between microbes within a sample and does not require rarefaction. Betta function of breakaway package [84,85] was used to compare alpha metrics between studied groups. Betta is a high-powered method for testing heterogeneity of total diversity. It accounts for missing taxa and does not require rarefaction. Compositional beta (between-sample) diversity was estimated using decode plugin for QIIME2 [86]. DEICODE method utilizes a form of Aitchison Distance that is robust to high levels of sparsity. Based on DEICODE distance, differences in centroids (multivariate means) of experimental groups were analyzed using Permutational Multivariate Analysis of Variance (PERMANOVA), as implemented in adonis function of vegan R package [87]. PERMANOVA’s assumption of equal dispersions among groups was validated using betadisp function of vegan package, followed by ANOVA. Pairwise PERMANOVA was performed using pairwiseAdonis R package [88]. pairwiseAdonis: Pairwise multilevel comparison using adonis. R package version 0.4) with Bonferroni adjustment for multiple comparisons. DEICODE distances between samples were visualized with 1) Principal Coordinate Analysis (PCoA) via ordinate function of phyloseq R package (REF: phyloseq: An R package for reproducible interactive analysis and graphics of microbiome census data) and 2) dendrogram generated using data from average linkage hierarchical clustering and ggdendro R package [89]. Differentially abundant ASVs were identified using the Wald or LRT method of DeSeq2 R package [90] and *p*-values were adjusted for multiple comparisons using the Benjamini and Hochberg method [91]. Prior to this analysis step, the dataset was filtered to remove ASVs with a total frequency of less than 10 and was found in less than 33% of samples. Although DeSeq2 tool is not dedicated for metagenomic data, it was shown to perform very well for metagenomic studies with small sample sizes, such as our own [92].

## 5. Conclusions

We found that the oral supplementation of suckling piglets with iron oxide nanoparticles for a period of 23 consecutive days results in a minor therapeutic effect compared to the administration of Sucrosomial^®^ or FeDex iron, which is correlated with reduced parameters of piglet growth. Although the administration of IONPs seems to be a low-toxicity procedure for both piglets and their microbiota, the daily dose of 6 mg of Fe used in the study does not allow the involvement of this compound into supplementation strategies. In pig breeding, even a minimal impairment of daily weight gain in the postnatal period may bring measurable financial losses during fattening to breeders.

## Figures and Tables

**Table 1:** Red blood cell (RBC) indices of supplemented piglets.

Parameter	Groups								
	No Treatment	FeDex	Sucrosomial^®^ Iron	IONP’s Phospholipid	Synomag^®^	IONP’s Dextran	$FeSO_{4}$	F	*p*
**RBC** (×10^6^/µL)	3.96	6.35 ***	6.06 **	5.84 **	5.31	4.86	5.51 *	4.20	0.0028
**RBC-He** (pg)	11.2	14.3 **	13.0	11.0	11.4	10.9	11.9	5.66	<0.0001
**HGB** (g/dL)	4.40	9.15 ***	7.88 ***	6.42	6.07	5.37	6.58 *	8.23	<0.0001
**HCT** (%)	12.7	29.2 ***	24.8 ***	20.4 *	19.5	16.6	21.4 *	8.10	<0.0001
**MCV** (fL)	32.0	45.8 ***	41.1 *	34.9	36.7	34.2	38.6	5.87	0.0003
**RDW** (%)	46.6	35.7 *	37.3 *	46.2	45.4	45.2	41.1	3.76	0.0056
**MCH** (pg)	11.1	14.4 ***	13.1 *	11.0	11.4	11.0	12.1	7.49	<0.001
**RET** (K/uL)	18.2	56.9	115 **	8.97	18.7	10.9	36.4	5.59	<0.001
**RET-He** (pg)	15.6	14.6	16.1	18.6	17.4	13.9	13.4	1.24	0.310

Table 1. Blood cell indices were determined for piglets from each group on day 28 after birth: Red blood cell count (RBC); Red blood cell hemoglobin content (RBC-He); hemoglobin concentration (HGB); hematocrit value (HCT); red cell distribution width (RDW); mean corpuscular volume (MCV); mean cell hemoglobin (MCH); reticulocytes count (RET); reticulocytes hemoglobin content (RET-He). Data are presented as the mean ± SEM, (*n* = 6), * and ** or *** asterisks denote statistically significant differences at *p* < 0.05 and *p* < 0.01 or *p* < 0.001 in comparison to control, non-supplemented animals.

**Table 2:** Tissue and plasma iron content in supplemented groups.

	Tissue	Groups								
Parameter		No Treatment	FeDex	Sucrosomial^®^ Iron	IONP’s Phospholipid	Synomag^®^	IONP’s Dextran	$FeSO_{4}$	F	*p*
**Non-heme iron level** **(mg Fe/kg of wet tissue)**	**liver**	35.6	112 ***	32.4	22.9	25.0	29.0	33.6	15.2	< 0.0001
	**spleen**	94.8	46.4 *	51.9 *	65.9	57.8	57.1	52.1	2.01	0.0927
**Plasma iron level** **(ug/dL)**		18.3	35.1 *	31.1 *	20.1	14.9	12.9	20.9	1.61	0.1779
**TIBC** **(ug/dL)**		403	350	365	354	363	448	338	0.867	0.5291
**TSAT** **%**		19.2	34.4	23.5	27.2	27.5	20.8	30.4	1.20	0.3297

Table 2. Animals supplemented with SI but not IONPs show increased blood plasma iron level in comparison to control animals and no excessive iron accumulation in liver and spleen is visible in the case of nano-iron supplementation in comparison to controls: Total iron binding capacity (TIBC); transferrin saturation (TSAT). Data are presented as the mean ± SEM, (*n* = 6), * and *** asterisks denote statistically significant differences at *p* < 0.05 and *p* < 0.001 in comparison to control, non-supplemented animals.

## Supplementary Materials

The following are available online at https://www.mdpi.com/article/10.3390/ijms22189930/s1, Figure S1: The role of oral iron supplementation in modulation of piglets microbiome., Table S1: Parenteral administration of iron shows a significant advantage over oral administration in terms of piglet growth., Table S2: The composition and chemical analysis of the basal diet of pregnant sows. (g/kg as-fed basis if not stated otherwise), Table S3: Real Time PCR primers used in the study., Table S4: Antibodies used in Western-Blot analyses., Supplemenetary Materials and Methods and files with raw data.
