# Supplementary material for: Comparative Evaluation of Sucrosomial Iron and Iron Oxide Nanoparticles as Oral Supplements in Iron Deficiency Anemia in Piglets
Source: Int J Mol Sci. 2021 Sep 14;22(18):9930. doi: 10.3390/ijms22189930 (PMC8466487; doi:10.3390/ijms22189930)

Supplementary materials.

Supplementary Tables.

Supplementary Table S1. Parenteral administration of iron shows a significant advantage over oral administration in terms of piglet growth.

Parameter	Groups							F	P
	Anemia	FeDex	Sucrosomial® Iron	IONP's-Phospholipid	Synomag®	IONP's-Dextran	$FeSO_4$		
Initial BW (g)	1817	1890	1773	1727	1760	1762	1692	0.402	0.87
Final BW (g)	4980	7000 *	5917	6333	6167	6417	6583	1.65	0.1635
Average Daily Gain (g)	114	183 *	148	165	157	166	175	1.63	0.1685

Supplementary Table S1. Initial body weight of piglets; Final body weight of 28-days old piglets; Average daily gain (ADG) in piglets during the period between days 1 and 28 after birth; Values are expressed as the mean ± SEM, (n=6),), * asterisk denote statistically significant differences at $p < 0.05$ in comparison to control, non-supplemented animals

Supplementary Table S2. The composition and chemical analysis of the basal diet of pregnant sows. (g/kg as-fed basis if not stated otherwise)

**Ingredients (g / kg)**		**Chemicals composition (g / kg)**	
Ground barley	400.0	ME(MJ/kg)	13.7
Ground wheat	163.0	Dry matter	887.0
Ground maize	250.0	Crude protein	132.0
HP300[a]	100.0	Crude fat	71.7
Premix[b]	65,1	NDF	120.9
Calcium formate	15.2	ADF	40.3
Probiotics	0.7	Ash	45.0
NaCl	6.0	Phosphorus	6.2
		Calcium	8.3
Total	1000g	Fe(mg/kg)	40.0
		Cu(mg/kg)	3.0
		Zn(mg/kg)	15.0
		Mn(mg/kg)	30.0
		Lysine	9.9
		Methionine	2.9
		Threonine	6.8
		Tryptophan	1.8

Supplementary Table S2. [a] Soy protein concentrate. [b] Premix provided the following per kg of diets: Na 0.28 g, Ca 2.15 g, P 0.7 g; Mg 0.1 g; Lysine 0.2 g; Fe 37 mg: Mn 30 mg; I 0.75 mg; Zn 15 mg; Cu 2.55 mg; Co 0.1 mg; Se 0.1 mg, Methionine 50 mg; Threonine 90 mg, Tryptophan 15 mg, Histidine 17 mg, Vitamin A 10000 IU; Vitamin D3 1000 IU; Vitamin E 20 mg; Vitamin K3 1.5 mg; Vitamin B1 0.3 mg; Vitamin B2 0.8 mg; Vitamin B6 1.0 mg; Vitamin B12 10 µg; Niacin 5 mg; Folic acid 0.5 mg; Biotin 20 µg; Nicotinic acid 6.5 mg; Pantothenic acid 8 mg; Choline 25 mg. [c] Bonvital, lactic acid bacteria - Enterococcus faecium (DSM 7134, 4b1841), 1 x 10^{10} CFU/g,(SCHAUMANN Agri International GmbH).

Supplementary Table S3.
Real Time PCR primers used in the study.

Target gene	Forward primer (5′→3′)	Reverse primer (5′→3′)
	**Pig**	
**Fpn**	TCGCCTAGTGTCATGACCAG	CAGAAACACAGACACCGCAA
**DMT1**	GCAGGTGGTTGACGTCTGTA	CACGCCCCCTTTGTAGATGT
**HPRT**	GGCCATCACATCGTAGCCCT	TCGCCCGTTGACTGGTCATT
**LfT**	TGTTTGGACGGAACAGACCC	CTCATGGCTGGTCGGCAATA
**Hepc**	ATCCCAGACAAGACAGCTCACA	TCTTGCAGCACATCCCACAGA
**RPL4**	CTCAGCGAATGAGAGCTGGT	GTGTTTCGGCGCATTGTCTT
	**Human**	
**Fpn**	CAAAAGCCACACAGAAGGGC	AGCAACGTATTGCAGTCTCC
**DMT1**	TATGTGCCGTTGCACAGACT	TAAACTGAGCTGGCCCTTGG
**TfR1**	GGACGCGCTAGTGTTCTTCT	CATCTACTTGCCGAGCCAGG
**HPRT**	TTGCTTTCCTTGGTCAGGCA	ATCCAACACTTCGTGGGGTC
**LfT**	CCAGCACCGTTTTTGTGGTT	CAATTCGCGGAAGAAGTGGC
	**Mouse**	
**Fpn**	TTGCAGGAGTCATTGCTGCTA	TGGAGTTCTGCACACCATTGA
**DMT1**	CTCCACCATGACTGGAACC	TTCAGGAATCCCTCCATGAC
**TfR1**	CCATGTTTTGACCAATGCTG	TCGCTTATATTGGGCAGA CC
**B-Actin**	TCCCTGGAGAAGAGCTATGA	AGCACTGTGTTGGCATAGAG
**HfT**	CAGGGTGTGCTTGTCAAAGA	GCTGAATGCAATGGAGTGTG

Supplementary Table S4.
Antibodies used in Western-Blot analyses.

Target protein	Primary Ab	Primary Ab dilution	Secondary Ab	Secondary Ab dilution
**TfR1**	Transferrin receptor monoclonal antibody Mouse monoclonal, Thermo Fisher #136800	1:1000	Goat anti-mouse polyclonal, #A5278 (Sigma-Aldrich)	1:10000
**Fpn**	Rabbit Anti-Mouse Metal Transporter Protein1/Ferroportin (MTP1/IREG1/Fpn) IgG aff. pure #MTP11-A Alpha Diagnostic Intl. Inc	1:1000	Goat anti-rabbit polyclonal, #A6154 (Sigma-Aldrich)	1:10000
**DMT1**	Anti-Human Natural Resistance-Associated Protein 2 (DMT1/NRAMP2, with IRE) #NRAMP22-A Alpha Diagnostic Intl. Inc	1:1000	Goat anti-rabbit polyclonal, #A6154 (Sigma-Aldrich)	1:10000
**Lft**	Rabbit polyclonal Anti-Ferritin Light Chain antibody ab69090 ABCAM	1:1000	Goat anti-rabbit polyclonal, #A6154 (Sigma-Aldrich)	1:10000
**Hft**	Rabbit polyclonal Anti-Ferritin Heavy Chain antibody ab65080 ABCAM	1:1000	Goat anti-rabbit polyclonal, #A6154 (Sigma-Aldrich)	1:10000
**B-Actin**	beta Actin Loading Control Monoclonal Antibody (BA3R) Invitrogen	1:1000	Goat anti-mouse polyclonal, #A5278 (Sigma-Aldrich)	1:10000
**Hx**	Rabbit polyclonal Anti-Hemopexin antibody HPEX16-A AlfaDiagnostics	1:1000	Goat anti-rabbit polyclonal, #A6154 (Sigma-Aldrich)	1:10000
**Hp**	Chicken polyclonal Anti-Haptoglobin antibody H1820-07 MSBiological	1:1000	Goat anti-chicken polyclonal, SAB3700195(Sigma-Aldrich)	1:10000
**Alb**	Rabbit polyclonal Anti-Albumin, sc-50536 Santa Cruz Biotechnology	1:1000	Goat anti-rabbit polyclonal, #A6154 (Sigma-Aldrich)	1:10000

Supplemenetary Materials and Methods.

1. ***Metagenomic analysis.***

1.1. ***Raw data generation***

For the sequencing experiment, 34 fecal samples (4-6 samples per group) were collected from piglets belonging to experimental groups. To minimize the litter effect, care was taken to distribute piglets from a single mother equally between groups. Samples used for the sequencing are described in detail in supplementary materials (metadata_enhanced.tsv). DNA libraries were prepared according to the Illumina 16S metagenomic library preparation protocol. Q5 Hot Start High-Fidelity polymerase (NEB, UK) and 341F/785R primer pair were used for amplification of V3-V4 hypervariable region of 16S rRNA gene. Paired-end, 2 x 300 bp-long reads were generated using MiSeq device and MiSeq Reagent Kit v3 (Illumina, USA). Samples were demultiplexed and fastq files containing raw data were generated using MiSeq Reporter (MSR) v2.6 software (Illumina, USA). Quality control of raw data was performed using fastqc v0.11.9 tool (REF: FastQC: a quality control tool for high throughput sequence data). All procedures were performed according to manufacturer's protocols, unless stated otherwise.

1.2. ***Data pre-processing***

Data pre-processing was performed on QIIME2 platform (REF: Reproducible, interactive, scalable and extensible microbiome data science using QIIME 2) and set up into a pipeline using nextflow (REF: Nextflow enables reproducible computational workflows). All processed data can be found in Supplementary Data (preprocessed_data), while quality control files in Supplementary Data (preprocessed_data_qa). Identical reads were merged into Amplicon Sequence Variants (ASV) using DADA2 denoising tool (DADA2: High resolution sample inference from Illumina amplicon data). This method was used instead of Operational Taxonomic Units (OTU) clustering due to its reproducibility as well as abilities to capture all biological variation present in the data and to better discriminate between taxa (REF: exact sequence variants should replace operational taxonomic units in marker-gene data analysis). As a part of denoising step, reads were trimmed from the start position to remove primer sequences. Trimming length was equal to length of 341F (17 bases, forward read) and 785R (21 bases, reverse read) primers. Additionally, reads were truncated (shortened) from the end position, to remove poor quality bases and maximize number of reads surviving denoising. Optimal truncation parameters (268 for forward read and 218 for reverse read with maximal expected error rate of 2) were established using Figaro tool (REF: FIGARO: An efficient and objective tool for optimizing microbiome rRNA gene trimming parameters). Pseudo-pooling method was used for DADA2, as it allows to retain some of the singletons, which is crucial for downstream analysis. ASVs were placed on phylogenetic tree using fragment-insertion sepp plugin for QIIME2 (REF: Phylogenetic Placement of Exact Amplicon Sequences

Improves Associations with Clinical Information) and Silva 128 SEPP reference database provided by QIIME2 team. Taxonomy data were extracted from SILVA 138.1 SSU NR99 database[The SILVA ribosomal RNA gene database project: improved data processing and web-based tools] and assigned to ASVs using classify-consensus-vsearch plugin for QIIME2.

2. Sucrosomial® Iron composition.

INGREDIENTS: - Ferro Sucrosomiale® (iron pyrophosphate – 6 mg of Fe/g of powder, pregelatinised rice starch. Saccharic esters of fatty acids, sunflower lecithin supported on glucose syrup, milk proteins, tricalcium phosphate)

Supplementary Figures.

Supplementary Figure S1.

The role of oral iron supplementation in modulation of piglets microbiome.

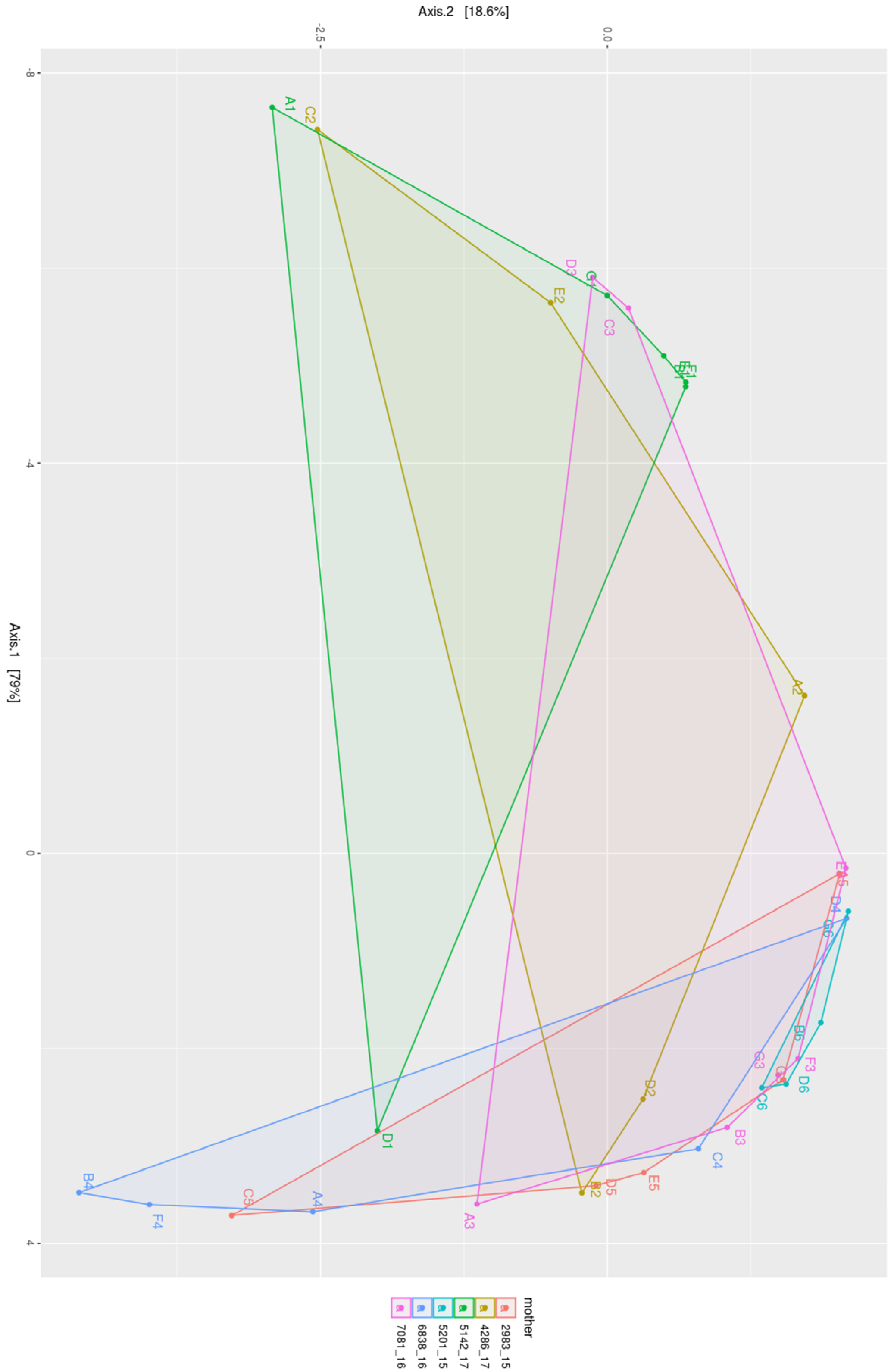

Supplement: Supplementary file 1 [file ijms-22-09930-s001.zip › Supplementary-MaterialsRRS-sub.pdf]
