# Supplementary figures and images for: Comparative Evaluation of Sucrosomial Iron and Iron Oxide Nanoparticles as Oral Supplements in Iron Deficiency Anemia in Piglets
Source: Int J Mol Sci. 2021 Sep 14;22(18):9930. doi: 10.3390/ijms22189930 (PMC8466487; doi:10.3390/ijms22189930)

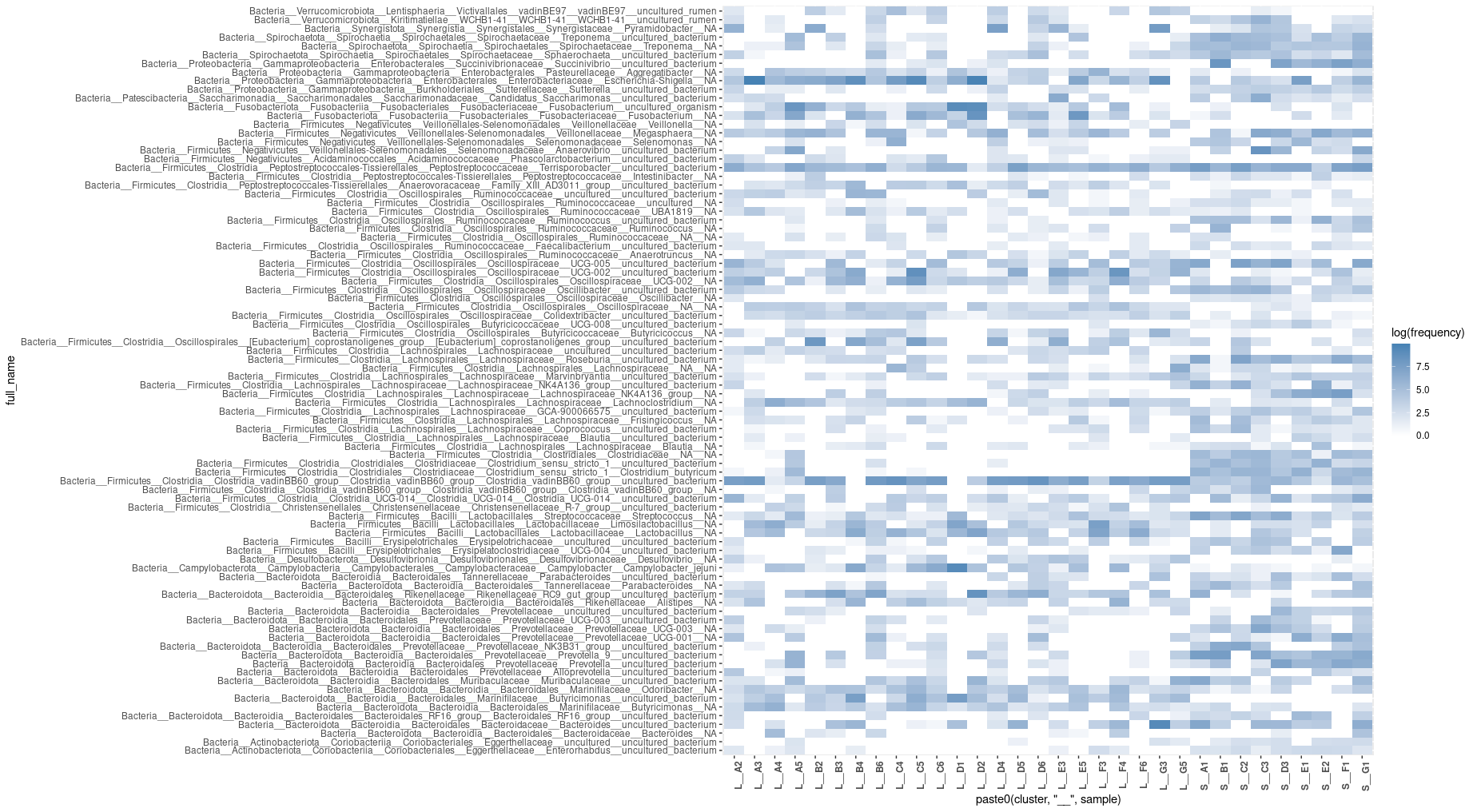

Supplement: Supplementary file 1 [file ijms-22-09930-s001.zip › sup_tables/cluster_heatmap.png]

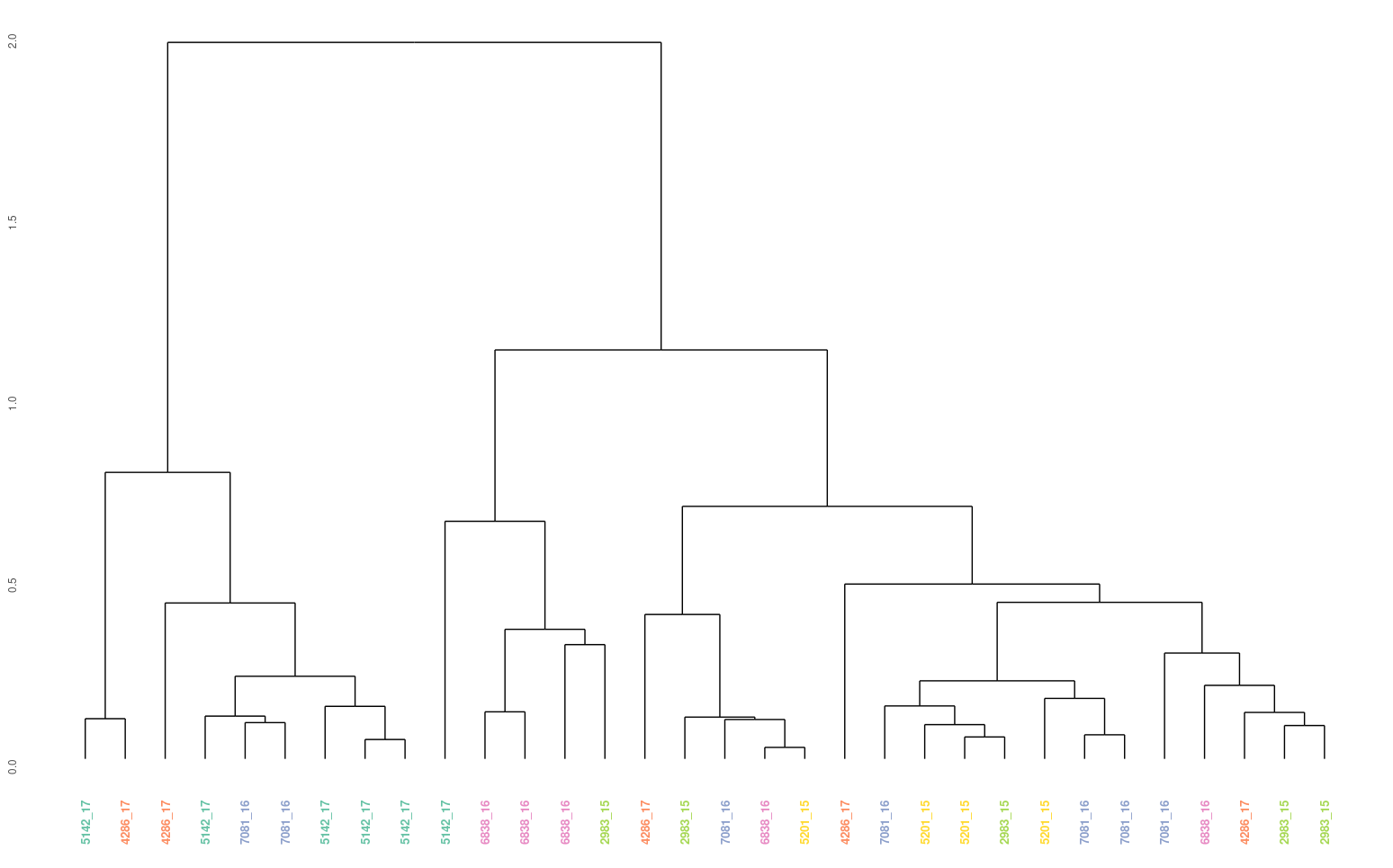

Supplement: Supplementary file 1 [file ijms-22-09930-s001.zip › sup_tables/dendrogram_mother.png]

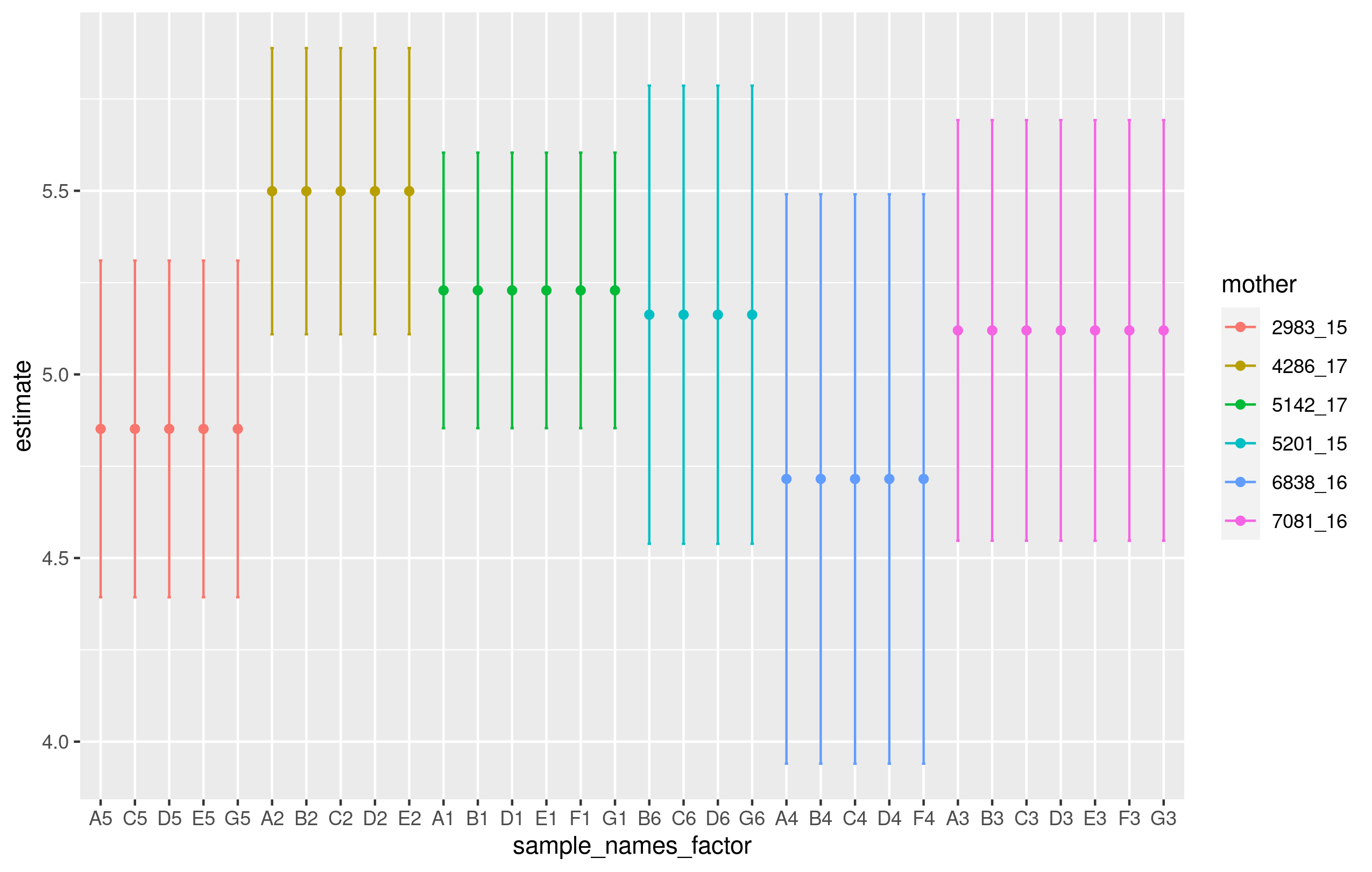

Supplement: Supplementary file 1 [file ijms-22-09930-s001.zip › sup_tables/mother____shannon__mother.png]

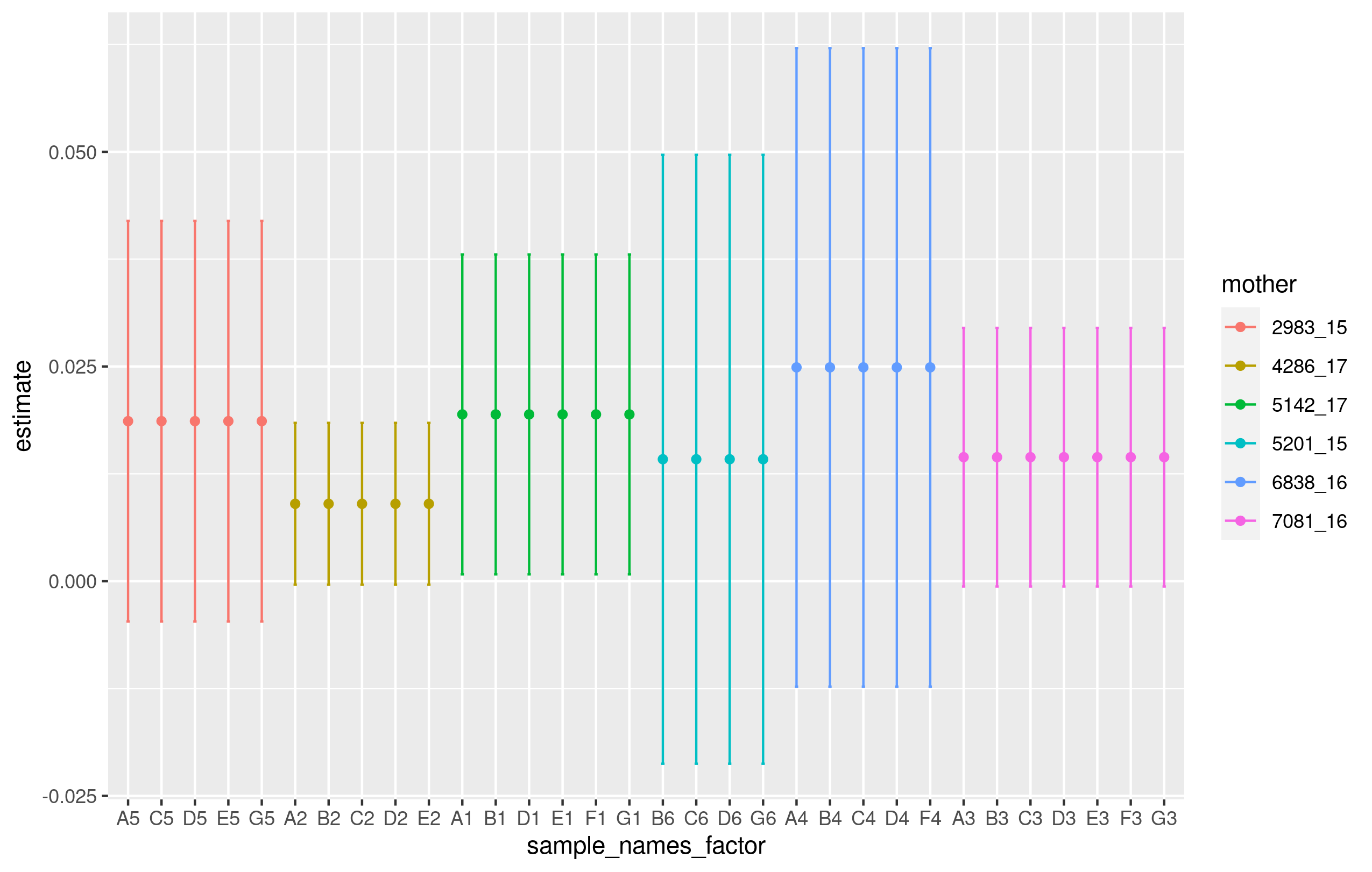

Supplement: Supplementary file 1 [file ijms-22-09930-s001.zip › sup_tables/mother____simpson__mother.png]

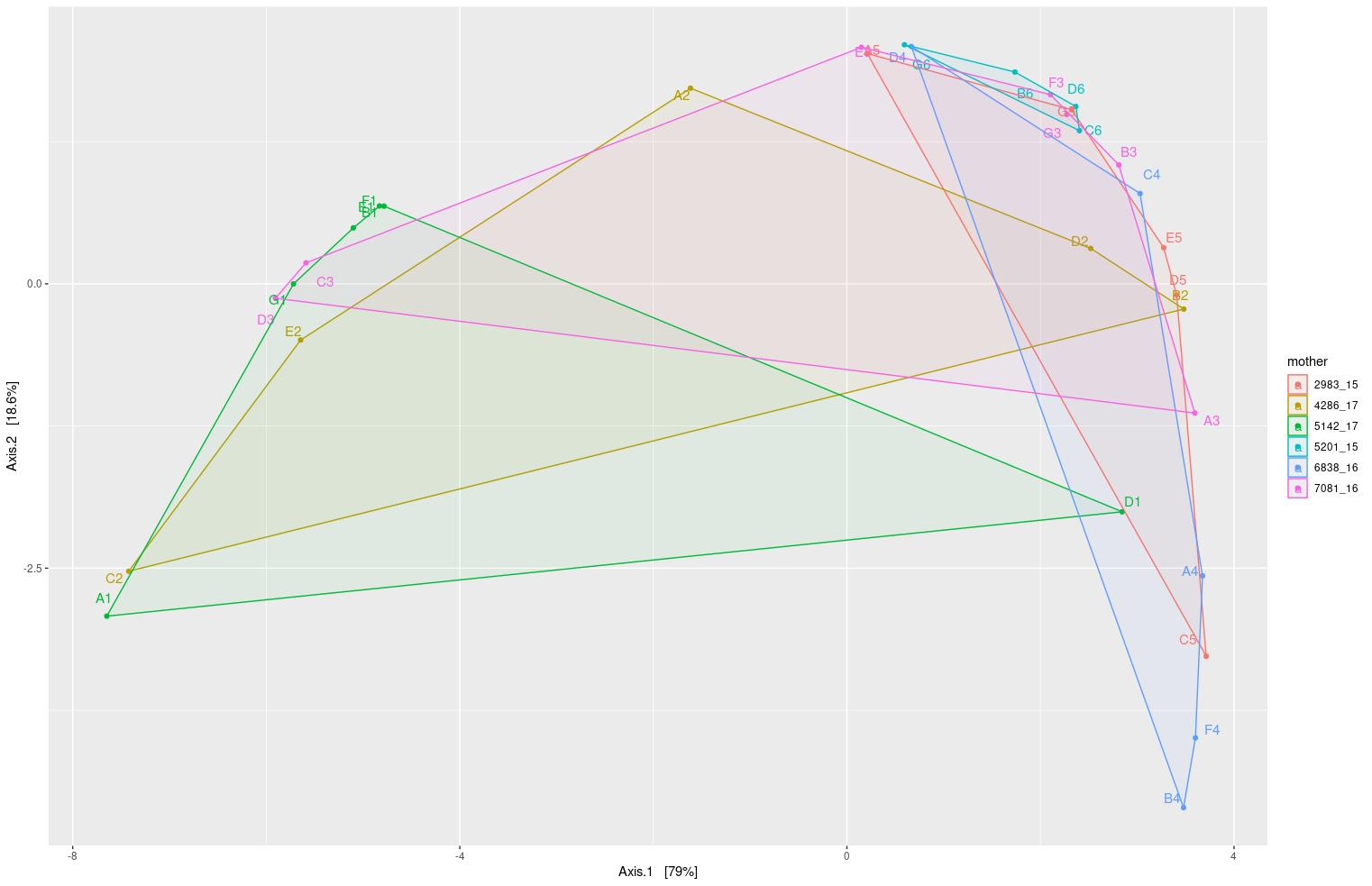

Supplement: Supplementary file 1 [file ijms-22-09930-s001.zip › sup_tables/pcoa_mother.png]

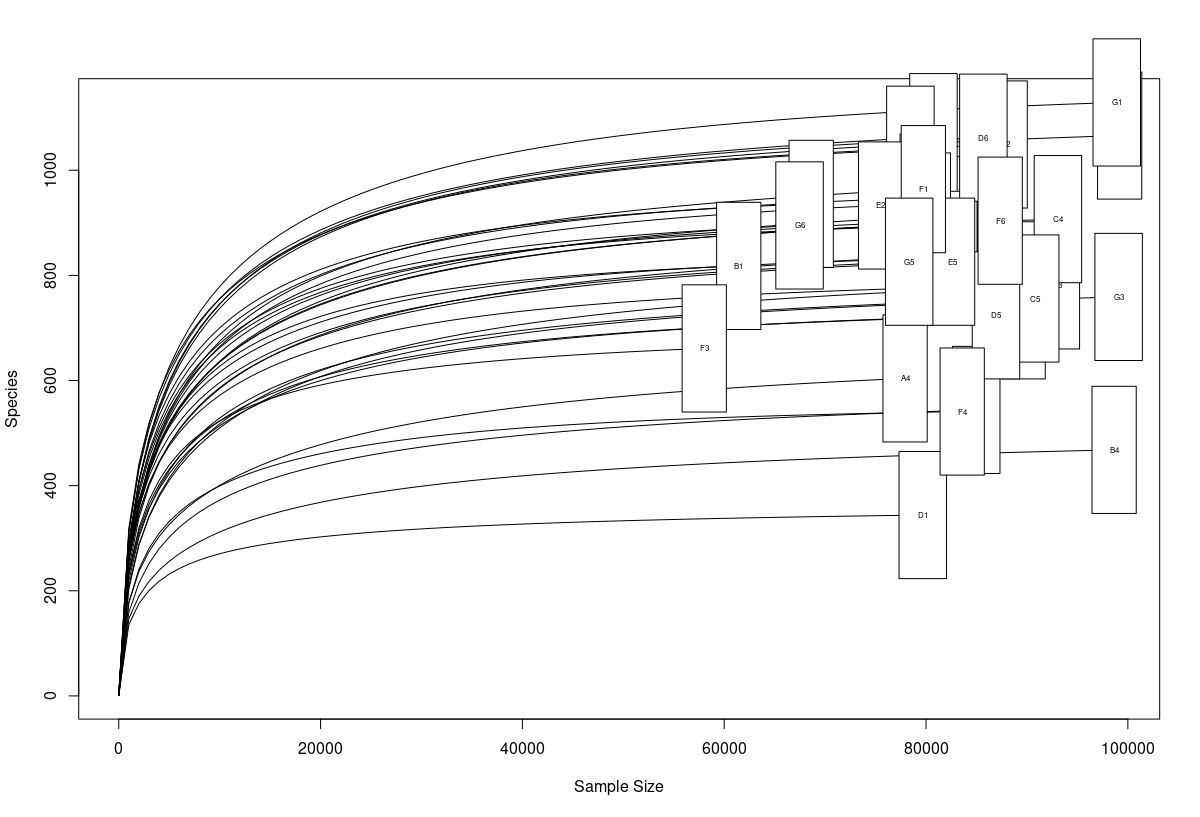

Supplement: Supplementary file 1 [file ijms-22-09930-s001.zip › sup_tables/rarefaction_curve.png]
